# Chirurgie bei Patienten mit Leberzirrhose

**DOI:** 10.1007/s00104-020-01319-z

**Published:** 2021-01-18

**Authors:** Roxana Pantea, Phil Meister, Jan P. Neuhaus, Knut Nowak, Andreas Paul, Fuat H. Saner

**Affiliations:** Klinik für Allgemein‑, Viszeral-, und Transplantationschirurgie, Universitätsklinikum Essen, Hufelandstr 55, 45147 Essen, Deutschland

**Keywords:** MELD, Mortalität, Komplikationsrate, Krankenhausletalität, Postoperative Morbidität, MELD, Mortality, Complication rate, Hospital mortality, Postoperative morbidity

## Abstract

**Hintergrund und Ziel der Arbeit:**

Patienten mit einer Leberzirrhose, die eine operative Behandlung benötigen, weisen eine hohe Krankenhausmortalität auf. Die vorliegende Studie untersucht die postoperative Morbidität sowie Krankenhausmortalität nach stattgehabter Operation von Patienten mit einer Leberzirrhose.

**Material und Methode:**

Retrospektiv wurden im Zeitraum von 01/2010 bis 12/2017 321 Patienten mit einer Leberzirrhose in unserer Klinik operativ behandelt. Erfasst wurden leberspezifische Scoringsysteme wie MELD(Model of End Stage Liver Disease)- und Child-Pugh-Score (CPS), die Krankenhausletalität und die postoperative Morbidität wurden mittels der Dindo-Clavien-Klassifikation erhoben.

**Ergebnisse:**

Von den 321 Patienten (68 % männlich) wurden 21,2 % als Notfall versorgt. Die Letalität der Notfallpatienten war mit 60 % signifikant höher als die der elektiv operierten Patienten (12 %, *p* < 0,0001). Komplexe Eingriffe zeigen insgesamt eine Letalität von 41 %, kleinere Eingriffe immer noch 20,5 % (*p* = 0,0001). Die postoperativen Komplikationsrate und Mortalität zeigten sich nach CPS signifikant unterschiedlich bei 11,8 % bzw. 6,3 % in der CPS-A-Kategorie im Vergleich zu 84 % bzw. 73 % in der CPS-C-Kategorie (*p* = 0,001). Statistisch steigt die Krankenhausletalität um etwa 20 % mit jedem MELD-Anstieg um einen Punkt (OR 1,23, *p* = 0,0001). Am schwerwiegendsten ist das Vorliegen einer hepatischen Dekompensation.

**Diskussion:**

Operative Eingriffe von Patienten mit Leberzirrhose sind mit einer hohen Komplikationsrate und Krankenhausletalität verbunden. CPS und MELD können bei der objektiven Risikoeinschätzung helfen, während auch die klinische Untersuchung auf Zeichen einer hepatischen Dekompensation von Bedeutung ist. Natrium, Kreatinin und andere Laborwerte können diese Einschätzung ergänzen.

## Abstract (en)

**Background and objective:**

Patients with liver cirrhosis who are in need of surgery show a high in-hospital mortality. This study examined the risk of postoperative morbidity and in-hospital mortality after operations on patients with liver cirrhosis.

**Material and methods:**

In the time period from January 2010 to December 2017 a total of 321 patients with liver cirrhosis underwent general surgery in this department. Liver-specific scoring systems, such as the Child-Pugh score (CPS) and MELD score were retrospectively assessed and correlated with in-hospital mortality and perioperative morbidity using the Dindo-Clavien classification.

**Results:**

Of the 321 patients (68% male) 21.2% underwent emergency surgery. These patients showed a mortality of 60%, which is significantly higher than in elective surgery (12%, *p* < 0.0001). Complex interventions showed a 41% mortality, minor interventions still 20.5% (*p* = 0.0001). The postoperative complication rate and mortality were significantly different sorted by CPS with 11.8%/6.3% in the CPS A category and 84%/73% in the CPS C category, respectively (*p* = 0.001). Statistically in-hospital mortality was increased by 20% for every point increase in the MELD score (odds ratio, OR 1.23, *p* = 0.0001). The presence of hepatic decompensation had the worst prognosis.

**Conclusion:**

Surgical interventions in patients with liver cirrhosis are associated with a high complication rate and in-hospital mortality. The CPS and MELD scores can be used for objective risk assessment, while clinical examination for signs of hepatic decompensation is also important. Laboratory values, such as sodium and creatinine can assist the assessment.

## Hintergrund

Aufgrund des besseren Verständnisses der Pathophysiologie sowie Therapie können Patienten mit einer Leberzirrhose auch über einen längeren Zeitraum gut kompensiert leben [1]. Die Entwicklung von Aszites, das Auftreten einer hepatischen Enzephalopathie, gastrointestinaler Blutungen, eines nichtmechanischen Ikterus oder bakterieller Infektionen führen zu akuter Dekompensation (AD; [2]). Eine AD kann sowohl rekompensieren, aber auch weiter zu einem „acute-on-chronic liver failure“ (ACLF, akut-auf-chronisches Leberversagen) mit einem oder Mehrorganversagen führen [3]. Die Prävalenz der Leberzirrhose ist nicht exakt bekannt, scheint regional unterschiedlich zu sein (bis zu 10 %) und ist nur in 75 % der Fälle präoperativ gesichert [4]. Operative Eingriffe erfolgen sehr häufig in den letzten beiden Lebensjahren, weshalb die Morbidität und Mortalität mutmaßlich in der bisherigen Publikation hoch ist [5].

Zur Abschätzung der Leberfunktion werden 2 Scoringsysteme verwendet:

- der MELD(Model of End Stage Liver Disease)-Score und
- der Child-Pugh-Score (CPS).

Der Chirurg Charles Gardner Child (mit Jeremiah G. Turcotte) schlug den CPS erstmals 1964 vor, um die perioperative Mortalität von Patienten mit einer Zirrhose abzuschätzen. Pugh et al. modifizierte den Score im Jahr 1973 hinsichtlich der chirurgischen Behandlung von Blutungen aus Ösophagusvarizen [6]. Der MELD-Score wurde von Kamath et al. [7] zur Prognoseeinschätzung hinsichtlich des 3‑Monats-Überlebens von Patienten mit einer Leberzirrhose vorgestellt. In der Literatur finden sich Hinweise, dass der MELD gegenüber dem CPS besser geeignet ist, um die Prognose bei intraabdominellen Eingriffen abzuschätzen [8]. In einer Folgearbeit konnten Teh et al. [9] zeigen, dass ein präoperativer MELD-Score über 20 mit einer Krankenhausletalität von bis zu 50 % einhergeht.

Generell scheint es sich um ein Patientenkollektiv zu handeln, das trotz großer Sorgfalt bei der operativen und konservativen Therapie ein hohes Maß an Komplikationen bietet. In letzter Zeit wurde der MELD-Score für die Prognoseeinschätzung bei Leberzirrhose weiterentwickelt und der Natriumwert in den Score integriert [10, 11]. Ob der MELD-Na auch in Hinsicht auf die perioperative Prognose eine Verbesserung darstellt, ist bisher nicht bewertet.

Das Ziel unserer Arbeit war es, die Morbidität und Krankenhausletalität im eigenen Patientenkollektiv bei Elektiv- und Notfalleingriffen zu erfassen.

## Material- und Methode

Es handelt sich bei dieser Studie um eine retrospektive Untersuchung an Patienten mit einer Leberzirrhose, die in dem Zeitraum von Januar 2010 bis Dezember 2017 in unserer Klinik operativ behandelt wurden. Hierzu wurde die Einsicht und Auswertung vorhandener Patientendaten durch die Ethikkommission der Universität Duisburg-Essen genehmigt (Ethikantrag 18-8348-BO).

Die Patientendaten für die vorliegende Studie wurden aus den Krankenakten und aus dem Krankenhausinformationssystem entnommen.

Die Diagnose der Leberzirrhose galt als gesichert, wenn diese durch eine Leberbiopsie nachgewiesen war oder im Rahmen eines bildgebenden Verfahrens fachradiologisch diagnostiziert wurde. Je nach Ausmaß des Eingriffs wurden die Operationen in drei Kategorien sortiert:

- In die Kategorie Minor (*n* = 122) fielen weniger komplexe Eingriffe, wie Appendektomien, Hernieneingriffe, kleinere Dünndarmresektionen und explorative Laparotomien ohne wesentliche Organresektionen.
- Entsprechend fielen komplexere Eingriffe, wie onkologische Resektionen etc., in die Kategorie Major (*n* = 90).
- Alle Leberresektionen wurden in einer eigenen Kategorie sortiert (*n* = 109).

Ferner wurden die elektiven Eingriffe (*n* = 256) genauer eingeteilt und die Gruppen Hernieneingriffe, Cholezystektomien, Leberresektionen, gastrointestinale Eingriffe (Operationen an Magen, Darm oder Speiseröhre) und andere geformt. In die letzte Kategorie fallen vor allem endokrin- und gefäßchirurgische Eingriffe sowie abdominalchirurgische Eingriffe, welche nicht in die anderen Kategorien passen.

Das Ausmaß der Leberinsuffizienz wurde präoperativ anhand der Scoringsysteme MELD, MELD-Natrium und Child-Pugh abgeschätzt. Neben den klinischen Scores wurden Laborparameter (Quick/INR[International Normalized Ratio], Albumin, Bilirubin, GOT [Glutamat-Oxalacetat-Transaminase], GPT [Glutamat-Pyruvat-Transaminase], Serumkreatinin, Fibrinogen, aPTT [aktivierte partielle Thromboplastinzeit], Thrombozyten) präoperativ erhoben. Komplikationen ab Dindo-Clavien Stufe 3 und die Krankenhausletalität wurden erfasst.

Die Auswertung und die graphische Darstellung erfolgte unter Verwendung von Excel 2019 (Microsoft Inc., Redmond WA, USA) und SPSS 25.0 (IBM Inc., Armonk NY, USA). Es wurden Analysen mit dem Student-t-Test sowie der binären logistischen Regression zur univariaten und multivariaten Analyse eingesetzt. Als signifikant wurden *p*-Werte kleiner *p* < 0,05 gewertet.

## Ergebnisse

### Patientencharakteristika

Insgesamt konnten 321 Patienten in diese Studie eingeschlossen werden. Hiervon waren mit 68,5 % der Großteil der Patienten männlichen Geschlechtes mit einem mittleren Alter von 63,9 Jahren. Der mittlere MELD-Score betrug 12,1, zum statistischen Vergleich wurden die Patienten nach niedrigem MELD (< 10, *n* = 144), mittlerem MELD (10–14, *n* = 105) und hohem MELD (>14, *n* = 69) gruppiert. 127 Patienten wiesen einen CPS A (39,6 %), 116 Patienten einen CPS B (36,1 %) und 50 Patienten einen CPS C (15,6 %) auf. Insgesamt wurden 256 elektive Operationen durchgeführt (79,8 %), die übrigen Eingriffe erfolgten als Notfalloperationen. Weitere Patientencharakteristika sind Tab. 1 zu entnehmen.

**Table 1:** 

*Alter (Jahre)*	63,94 ± 10,6 (28–92)
*Geschlecht (männlich)*	220 (68,5 %)
*Aszites*	182 (56,7 %)
*Hepatische Enzephalopathie*	27 (8,4 %)
*MELD*	12,09 ± 6,26 (6–40)
*MELD-Na*	13,58 ± 6,84 (6–40)
*Child-Pugh A*	127 (39,6 %)
*Child-Pugh B*	116 (36,1 %)
*Child-Pugh C*	50 (15,6 %)
*Minor (ohne Leberresektionen)*	122 (38 %)
*Major (ohne Leberresektionen)*	90 (28 %)
*Elektive Operationen:*	256 (79,8 %)
Hernienoperation (nur elektiv)	38 (14,8 %)
Gastrointestinale Operation (nur elektiv)	33 (12,9 %)
Cholezystektomie (nur elektiv)	34 (13,3 %)
Leberresektion (nur elektiv)	74 (28,9 %)
Andere (nur elektiv)	77 (30,1 %)

*MELD* Model of End Stage Liver Disease

^a^Angegeben ist die absolute Anzahl mit prozentualer Verteilung, bei Alter und MELD der Mittelwert mit Minimum, Maximum und Standardabweichung

### Morbidität und Mortalität

**Figure 1:**
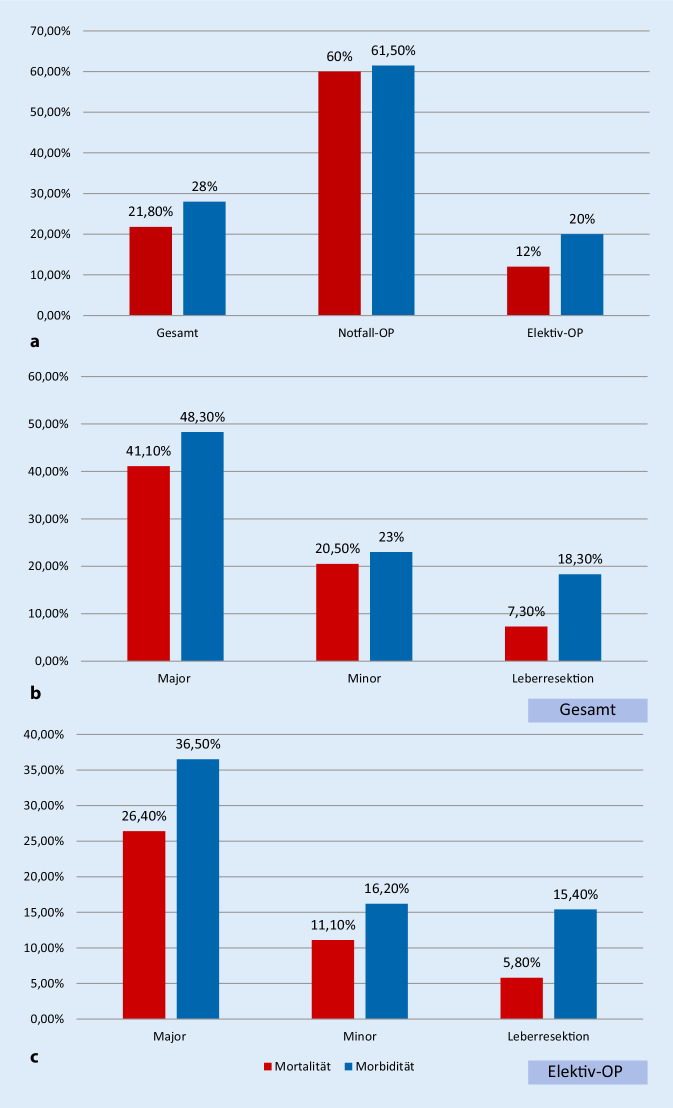

In unserem Patientenkollektiv betrug die gesamte Mortalität 21,8 % (*n* = 70). Nach einer notfallmäßigen Operation zeigte sich eine Mortalität von 60 %, während sie bei den elektiv operierten Patienten 12 % betrug (*p* < 0,001). Die Komplikationsrate bei den notfallmäßig operierten Patienten lag bei 61,5 % im Vergleich zu 20 % bei den Patienten, die elektiv operiert worden sind (*p* < 0,001). Im Rahmen von Major-Operationen kam es zu einer Sterblichkeit von 41,1 % und einer Komplikationsrate von 48,3 %, während es im Vergleich hierzu bei Minor-Operationen zu einer Sterblichkeit von 20,5 % und einer Komplikationsrate von 23 % kam. Leberresektionen liegen mit einer Sterblichkeit von 7,3 % bei einer Komplikationsrate von 18,3 % darunter (*p* = 0,001). Betrachtet man für diesen Aspekt lediglich die elektiven Operationen, gleichen sich die Raten etwas an: Major mit einer Sterblichkeit von 26,4 % und einer Komplikationsrate von 36,5 %, Minor mit einer Sterblichkeit von 11,1 % und 16,2 % Komplikationsrate sowie Leberresektionen mit 5,8 % Sterblichkeit und 15,4 % Komplikationsrate (*p* = 0,001). Relevante Unterschiede bei den mittleren MELD-Scores sind zu erwähnen: Major (11,1), Minor (11,4) und Leberresektion (9,2; *p* = 0,001; Abb. 1).

**Figure 2:**
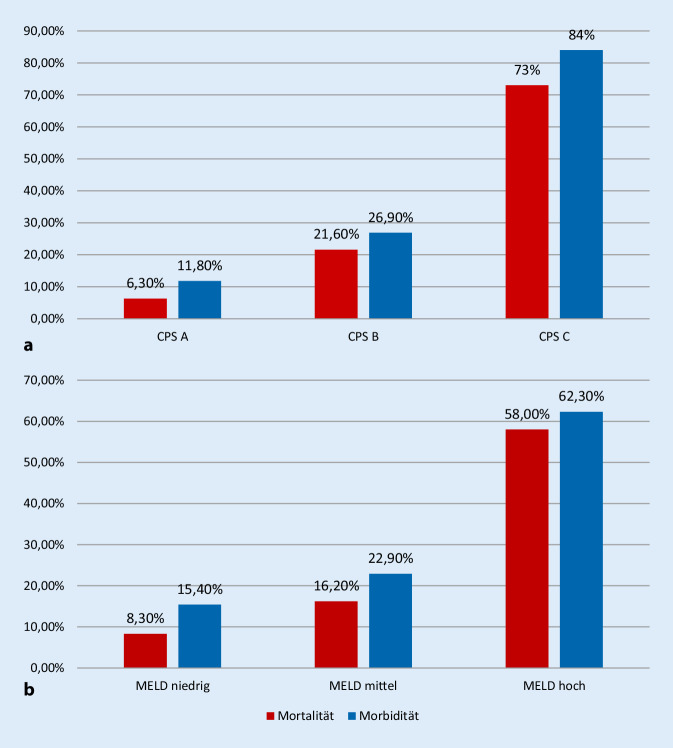

Die postoperative Komplikationsrate und Mortalität zeigten sich nach CPS signifikant unterschiedlich: bei 11,8 %, bzw. 6,8 % in der CPS-A-Kategorie, 26,9 %, bzw. 21,6 % in der CPS-B-Kategorie und 84 %, bzw. 73 % in der CPS-C-Kategorie (*p* = < 0,001). In den MELD-Gruppen zeigte sich bei niedrigem MELD eine Komplikationsrate von 15,4 % und eine Mortalität von 6,3 %, bei mittlerem MELD 22,9 % und 16,2 % und bei hohen hohem MELD 62,3 % und 58,8 % (*p* = < 0,001; Abb. 2).

Die Operationskategorie der elektiven Operationen hat ebenfalls einen signifikanten Einfluss auf die Mortalität und Komplikationsrate (*p* = 0,044). Die Operationen am Gastrointestinaltrakt zeigten die höchste postoperative Morbidität (37,5 %) und Mortalität (27 %), wobei die elektiven Cholezystektomien eine Morbidität von 8,8 % und eine Mortalität von 0 % zeigten.

Patienten mit einer Komplikation hatten durchschnittlich einen deutlich erhöhten MELD-Score (16,9 vs. 10,2 *p* = < 0,001), genauso wie Patienten, die verstarben (18,3 vs. 10,2 *p* = < 0,001). Die Patienten, die Komplikationen erlitten, hatten präoperativ niedrigere Quick- (62,29 % vs. 81,27 % *p* < 0,001) und Albuminwerte (26 g/l vs. 38 g/l *p* = 0,03) sowie höhere aPTT- (*p* < 0,001) und Bilirubinwerte (73,53 μmol/l vs. 22,23 μmol/l *p* < 0,001). Auch die Thrombozytenzahl (136,49 × 10^3^ vs. 175,44 × 10^3^ *p* = 0,04) und das Kreatinin (141,6 μmol/l vs. 105,315 μmol/l *p* = < 0,001) zeigten sich signifikant unterschiedlich bei Patienten mit Komplikationen.

Zum direkten Vergleich von MELD und MELD-Na zur Vorhersage der perioperativen Mortalität wurde eine ROC(„receiver operating characteristics“)-Kurve mit dem entsprechenden AUROC(„area under the receiver operating characteristic“)-Wert für beide Scores berechnet. Hierbei erreicht der normale MELD-Score einen AUROC-Wert von r = 0,802 und der MELD-Na-Score einen Wert von r = 0,838.

### Regressionsanalyse

Um die Bedeutung der einzelnen präoperativen Scores und Werte zu quantifizieren, wurde eine binär logistische Regressionsanalyse der relevant erscheinenden Faktoren durchgeführt (Tab. 2).

**Table 2:** 

	OR Komplikation	95 %-CI	*p*	OR Mortalität	95 %-CI	*p*
Alter	**0,95**	0,92–0,94	**0,0001**	**0,94**	0,91–0,97	**0,0001**
Geschlecht männlich	1,02	0,60–1,71	0,941	1,09	0,61–1,91	0,777
Notfalloperation	**6,40**	3,56–11,50	**0,0001**	**10,89**	5,84–20,28	**0,0001**
Aszites	**3,29**	1,85–5,84	**0,0001**	**7,07**	3,32–15,42	**0,0001**
Hepatische Enzephalopathie	**26,91**	7,85–92,1	**0,0001**	**29,61**	9,78–89,54	**0,0001**
CPS A	1	[Referenz]		1	[Referenz]	
CPS B	**2,76**	1,39–5,43	**0,0001**	**4,09**	1,76–9,48	**0,001**
CPS C	**39,20**	15,4–99,2	**0,0001**	**38,25**	14,86–98,43	**0,0001**
MELD <10	1	[Referenz]		1	[Referenz]	
MELD 10–14	1,63	0,85–3,10	0,137	2,16	0,96–4,66	0,06
MELD >14	**9,10**	4,67–17,70	**0,0001**	**15,17**	7,09–32,44	**0,0001**
MELD	**1,21**	1,14–1,27	**0,0001**	**1,23**	1,16–1,30	**0,0001**
MELD Natrium	**1,24**	1,17–1,30	**0,0001**	**1,20**	1,14–1,25	**0,0001**
Natrium	**0,82**	0,76–0,87	**0,0001**	0,87	0,82–0,92	0,0001
Quick (%)	**0,96**	0,94–0,96	**0,0001**	**0,94**	0,92–0,95	**0,0001**
aPTT (s)	**1,15**	1,09–1,20	**0,0001**	**1,16**	1,11–1,22	**0,0001**
Thrombozyten (×10^3^)	**0,99**	0,99–0,99	**0,005**	**0,99**	0,99–0,99	**0,003**
Kreatinin (μmol/l)	**144,255**	106,2–193,815	**0,0001**	**141,6**	105,315–187,62	**0,001**
Bilirubin (μmol/l)	**22,743**	20,178–25,65	**0,0001**	**22,572**	20,178–25,308	**0,0001**
Hepatozelluläre Karzinom	**0,36**	0,18–0,68	**0,002**	**0,43**	0,24–0,75	**0,003**
Arterieller Hypertonus	0,63	0,36–1,08	0,093	0,67	0,41–1,10	0,116
Koronare Herzkrankheit	0,44	0,14–1,28	0,132	0,61	0,25–1,44	0,257
Diabetes	1,58	0,88–2,80	0,123	0,76	0,45–1,27	0,305
Niereninsuffizienz dialysepflichtig	1,82	0,16–20,41	0,626	1,27	0,84–14,17	0,846
*Operationsart*						
Minor	1	[Referenz]		1	[Referenz]	
Major	**2,71**	1,47–4,97	**0,001**	**3,14**	1,73–5,67	**0,0001**
Leberresektion	**0,31**	0,13–0,71	**0,006**	0,75	0,39–1,43	0,390
Hernie	1	[Referenz]		1	[Referenz]	
Cholezystektomie	0,43	0,10–1,81	0,249	^b^	^b^	^b^
Gastrointestinal	2,66	0,89–7,89	0,078	2,48	0,73–8,32	0,143
Leberresektion	1,13	0,42–3,05	0,816	0,48	0,12–1,76	0,269
Sonstige	0,98	0,36–2,66	0,975	1,22	0,39–3,75	0,730

*aPTT* aktivierte partielle Thromboplastinzeit,* CPS* Child-Pugh-Score, *MELD* Model of End Stage Liver Disease

^a^Statistisch relevante Ergebnisse sind **fett** hervorgehoben: Bei einem Odds Ratio (*OR*) von 1 ist kein Zusammenhang zu erwarten, eine negative Odds Ratio beschreibt eine Risikoreduktion, ein erhöhte Odds Ratio einen Anstieg des Risikos. Das Konfidenzintervall (*CI*) sollte nicht 1 umfassen, um als statistisch relevant zu gelten

^b^Die Krankenhausletalität bei den elektiven Cholezystektomien beträgt 0 %

Besonders hohen Einfluss haben die klinischen Parameter, wie das Vorliegen von Aszites (OR: 3,3 bzw. 7,1, *p* = 0,001) und hepatischer Enzephalopathie (26,9 bzw. 29,6, *p* = 0,001). Die Durchführung der Operation als Notfalloperation geht ebenfalls mit erhöhtem Risiko für Morbidität und Mortalität einher (OR: 6,4 bzw. 10,9). Im Vergleich der Eingriffsgröße gehen Major-Eingriffe mit einem erhöhten Risiko einher (OR: 3,1 bzw 2,7) als Minor-Eingriffe, wobei Leberresektionen ein geringeres Mortalitätsrisiko aufweisen (OR: 0,31).

Im Vergleich von Child-Pugh-Score und den MELD-Gruppen geht vor allem ein erhöhter CPS mit einem höheren operativen Risiko einher (OR: CPS B: 2,27 bzw. 4,09; CPS C: 39,2 bzw. 38,3), während erst ab einem MELD von 14 das Risiko statistisch relevant erhöht ist (OR: 9,1 bzw. 15,2). In Betrachtung allein der laborchemisch erhobenen Parameter sind vor allem ein erhöhter Kreatininwert (OR: 1,62 bzw. 1,60) und Bilirubinwert (OR: 1,33 bzw. 1,32) mit einer Erhöhung des Risikos verbunden. Die Risikoerhöhung durch einen erhöhten MELD-Wert liegt bei OR: 1,21/1,23. Zu beachten ist bei diesen metrischen Werten die lineare Erhöhung des Risikos durch Anstieg des Wertes. Der MELD-Na entspricht in seiner Risikoerhöhung in etwa dem konventionellen MELD (OR: 1,20/1,24).

Für die multivariate Analyse wurden drei Kategorien gebildet. In der ersten Kategorie wurden die klinischen Parameter (Alter, Notfalloperation, hepatische Dekompensation) gemeinsam mit dem MELD-Score analysiert. Hierbei gehen die Durchführung als Notfalleingriff und das Vorliegen einer hepatischen Dekompensation auch unabhängig vom MELD-Score mit einem erhöhten Risiko einher. In der nächsten Kategorie erfolgte der gemeinsame Vergleich von Eingriffsart mit MELD-Score. Hierbei ist eine Risikoerhöhung unabhängig vom MELD-Score auch durch die Durchführung eines Major-Eingriffs oder die Durchführung als Notfall zu notieren. Die zuvor beschriebene Risikosenkung bei einer Leberresektion ist in diesem Kontext nicht mehr nachweisbar. In der dritten Kategorie werden die Laborwerte miteinander verglichen, um voneinander unabhängige Risikofaktoren zu identifizieren. Hierbei ist vor allem ein erniedrigter Natriumwert unabhängig von den übrigen Laborwerten mit einem erhöhten Operationsrisiko verbunden (Tab. 3).

**Table 3:** 

	OR Komplikation	95 %-CI	*p*	OR Mortalität	95 %-CI	*p*
*Kategorie 1*						
Alter	0,98	0,94–1,02	0,392	0,98	0,95–1,01	0,255
Notfalloperation	**4,54**	2,03–10,12	**0,0001**	**2,78**	1,30–5,91	**0,008**
Aszites	**2,70**	1,08–6,73	**0,032**	1,26	0,63–2,47	0,512
Hepatische Enzephalopathie	**10,61**	3,04–36,93	**0,0001**	**10,20**	2,72–38,24	**0,001**
MELD	**1,11**	1,03–1,18	**0,003**	**1,11**	1,04–1,18	**0,001**
*Kategorie 2*						
Minor	1	[Referenz]		1	[Referenz]	
Major	1,58	0,75–3,30	0,227	**2,27**	1,12–4,57	**0,023**
Leberresektion	0,61	0,23–1,57	0,307	1,63	0,77–3,41	0,198
MELD	**1,16**	1,09–1,23	**0,0001**	**1,17**	1,10–1,24	**0,0001**
Notfalloperation	**4,08**	1,96–8,47	**0,0001**	**2,60**	1,26–5,31	**0,009**
*Kategorie 3*						
Natrium	**0,87**	0,80–0,93	**0,0001**	0,94	0,87–1,001	0,05
Quick (%)	**0,98**	0,95–0,99	**0,038**	0,99	0,96–1,004	0,128
aPTT (s)	**1,07**	1,01–1,13	**0,025**	**1,07**	1,01–1,13	**0,014**
Thrombozyten (×10^3^)	0,99	0,99–1,00	0,432	0,99	0,99–1,001	0,332
Kreatinin (μmol/l)	114,165	80,535–160,185	0,150	119,475	86,73–161,955	0,060
Bilirubin (μmol/l)	18,639	16,416–21,033	0,171	18,981	16,826–21,375	0,089

*aPTT* aktivierte partielle Thromboplastinzeit,* MELD* Model of End Stage Liver Disease

^a^Statistisch relevante Ergebnisse sind **fett** hervorgehoben: Bei einem Odds Ratio (*OR*) von 1 ist kein Zusammenhang zu erwarten, eine negative Odds Ratio beschreibt eine Risikoreduktion, eine erhöhte Odds Ratio einen Anstieg des Risikos. Das Konfidenzintervall (*CI*) sollte nicht 1 umfassen, um als statistisch relevant zu gelten

## Diskussion

Die chirurgische Therapie von Patienten mit Leberzirrhose stellt auch weiterhin eine Herausforderung für die behandelnden Ärzte dar. Die Letalität ist trotz aller Fortschritte in der operativen und perioperativen Medizin hoch. Noch vor 20 Jahren wurden Mortalitätsraten von 10 %, 32 %, 82 % beschrieben (klassifiziert nach CPS A, B und C; [12]). Diese Rate erscheint in unserem Patientenkollektiv mit 6,8 %, 21,6 % und 73 % nun reduziert.

In der vorliegenden Studie scheint der CPS das perioperative Risiko der Patienten besonders gut zu differenzieren. Uneinigkeit ergibt sich in der Bedeutung des MELD-Scores. Von vielen Autoren wird der MELD-Score als dem CPS überlegen betrachtet. So wird von einer deutlichen Risikoerhöhung bei einem MELD von über 14 [8] und auch einer linearen Abhängigkeit von MELD und perioperativer Mortalität [13] berichtet. Diese Zusammenhänge lassen sich an unserem Patientenkollektiv nur bedingt reproduzieren. Zwar weisen auch hier Patienten mit einem MELD-Score von über 14 eine deutlich erhöhte Morbidität und Mortalität auf, allerdings ist im direkten Vergleich der CPS überlegen. In der Regressionsanalyse wird die größte Risikoerhöhung (auch unabhängig vom MELD) durch das Vorliegen einer hepatischen Dekompensation, also konkret von Aszites und hepatischer Enzephalopathie, verursacht. Da der CPS diese Faktoren berücksichtigt, erscheint dessen Überlegenheit naheliegend. Von den „objektiv“ bestimmbaren (Labor‑)Werten geht vor allem ein erhöhter Kreatininwert als Einzelfaktor mit der höchsten perioperativen Letalität einher. Dies lässt sich dadurch erklären, dass bei Patienten mit Leberzirrhose ein Anstieg der Nierenretentionsparameter oft ein Zeichen für die kreislaufregulatorische Relevanz der Zirrhose ist.

Der Zusammenhang zwischen akuter Dekompensation und Organversagen, zumeist Nierenversagen, im operativen Setting wurde aktuell von Klein et al. beschrieben [14]. Hierbei wurde zentral das Vorliegen einer akuten Dekompensation zum Zeitpunkt einer Operation als entscheidender Risikofaktor für die Entwicklung eines „acute-on-chronic liver failures“ (ACLF) und damit für eine massive Prognoseverschlechterung beschrieben. Das neue und komplexe Krankheitsbild ACLF beschreibt vereinfacht zusammengefasst das gemeinsame Vorliegen einer akuten Dekompensation und einem (Multi‑)Organversagen, welches für die Prognose einer Leberzirrhose von großer Bedeutung ist.

In einer Metaanalyse von 118 Studien zum operativen Risiko bei Zirrhose wurde ausgearbeitet, dass der CPS bei Patienten ohne hepatische Dekompensation und der MELD bei Patienten mit Dekompensation zur Abschätzung der perioperativen Mortalität von Vorteil ist [15]. Diese Verallgemeinerung erfasst die Situation allerdings nur bedingt, da der CPS die Kriterien für eine akute Dekompensation selbst berücksichtigt.

Wir vergleichen erstmalig den konventionellen MELD-Score mit dem MELD-Natrium-Score zur Einschätzung der perioperativen Morbidität. Da das Serumnatrium auch in der multivariaten Analyse ein vom MELD-Score unabhängiger Risikofaktor ist, erscheint die Prüfung des MELD-Na-Scores gerechtfertigt. Tatsächlich ist auch dieser an unserem Patientenkollektiv für diesen Zweck verwendbar, der Zugewinn in der Vorhersage ist allenfalls marginal. Anhand unserer Daten sind beide Varianten des MELD-Scores am ehesten gleichwertig zu beurteilen.

Entscheidend erscheint anhand unserer Daten das Vorliegen einer hepatischen Dekompensation, wobei der CPS hier entsprechend hilfreich sein kann. Zur weiteren feineren Abschätzung können Parameter wie das Kreatinin, das Serumnatrium und der MELD-Score hinzugezogen werden. In der Literatur ist auch eine Risikoerhöhung durch eine erhöhte alkalische Phosphatase beschrieben [14], welche sich an unserem Patientenkollektiv nicht nachverfolgen lässt; dies ist möglicherweise in einem unterschiedlichen Anteil cholestatischer Lebererkrankungen begründet.

Auch der Eingriff selbst sollte in die Überlegungen bezüglich eines operativen Vorgehens mit einbezogen werden. Hernienreparaturen und Cholezystektomien gehen mit einem deutlich niedrigeren Risiko einher als komplexere abdominelle Eingriffe, wobei vor allem gastrointestinale Operationen risikobelastet erscheinen. So sind größere elektive Eingriffe mit einer Mortalität von 26 % verbunden, während kleinere elektive Eingriffe nur eine Mortalität von 11 % aufweisen. Passend hierzu konnte in der Untersuchung von Telem et al. auch bei Patienten mit CPS C eine geringe Mortalität von 12 % beschrieben werden, wobei die durchgeführten Operationen überwiegend mit geringem Trauma (Herniotomie) einhergingen [16].

Interessanterweise sind Leberresektionen in unserem Kollektiv nicht mit einem höheren Risiko verbunden als beispielsweise Hernieneingriffe, während sie in der in der Literatur oft als besonderer Risikofaktor beschrieben sind [5, 14]. Eine mögliche Erklärung ist eine besonders sorgfältige präoperative Selektion, welche sich auch an unterschiedlichen MELD-Scores der Patienten mit elektiven Minor-Eingriffen (11,4) und Leberresektionen (9,2) verdeutlichen lässt (*p* = 0,001). Passend hierzu gleicht sich das Risiko von Leberresektionen in der multivariaten Analyse unter Einbezug des MELD-Scores an. Nur durch die genaue Auswahl an geeigneten Patienten kann ein entsprechend niedriges Risiko bei Leberresektionen erreicht werden.

Mit der zunehmenden Bedeutung weiterer, oft nichtinvasiver Methoden zur Beurteilung von Leberfunktion und portaler Hypertension, wie der transienten Elastrographie von Leber und Milz oder der LiMAx®-Messung (Humedics GmbH, Berlin, Deutschland), ist deren Relevanz in der präoperativen Beurteilung von Patienten mit Leberzirrhose noch zu bestimmen, obwohl in der Leberchirurgie bei nichtzirrhotischen Patienten bereits ermutigende Ergebnisse vorliegen [17]. Bis dahin bleiben der CPS und die klinische Untersuchung bezüglich einer hepatischen Dekompensation die wichtigsten Instrumente zur Abschätzung des operativen Risikos bei Leberzirrhose.

## Fazit für die Praxis

- Operative Eingriffe bei Patienten mit Leberzirrhose sind mit einer hohen Komplikationsrate und Krankenhausmortalität verbunden. Besonders Notfalloperationen gehen mit einer ungünstigen Prognose einher.
- Entscheidend für die Risikoabschätzung ist der klinische Zustand und konkret das Vorliegen einer hepatischen Dekompensation.
- Child-Pugh- und MELD(Model of End Stage Liver Disease)-Score können bei der objektiven Risikoeinschätzung helfen.
- Serumnatrium, Kreatinin und andere Laborwerte können diese Einschätzung ergänzen.
- Der MELD-Na-Score ist gegenüber dem klassischen MELD in der perioperativen Risikoeinschätzung gleichwertig einzuschätzen.
- Wenig invasive Eingriffe können vergleichsweise sicher durchgeführt werden, während komplexe onkologische Eingriffe besonders risikobelastet sind.
